# Assessment of *TP53* lesions for p53 system functionality and drug resistance in multiple myeloma using an isogenic cell line model

**DOI:** 10.1038/s41598-019-54407-4

**Published:** 2019-12-02

**Authors:** Umair Munawar, Markus Roth, Santiago Barrio, Harald Wajant, Daniela Siegmund, Ralf C. Bargou, K. Martin Kortüm, Thorsten Stühmer

**Affiliations:** 1 Comprehensive Cancer Center Mainfranken, University Hospital of Würzburg, Würzburg, Germany; 2 Hospital 12 de Octubre, Madrid, Spain; 3 Division of Molecular Internal Medicine, Department of Internal Medicine II, University Hospital of Würzburg, Würzburg, Germany; 4 Department of Internal Medicine II, University Hospital of Würzburg, Würzburg, Germany

**Keywords:** Transposition, Myeloma

## Abstract

Recent advances in molecular diagnostics have shown that lesions affecting both copies of the gene for tumor suppressor protein 53 (*TP53*) count among the most powerful predictors for high-risk disease in multiple myeloma (MM). However, the functional relevance and potential therapeutic implications of single hits to *TP53* remain less well understood. Here, we have for the first time approximated the different constellations of mono- and bi-allelic *TP53* lesions observed in MM patients within the frame of a single MM cell line model and assessed their potential to disrupt p53 system functionality and to impart drug resistance. Both types of common first hit: point mutation with expression of mutant p53 protein or complete loss of contribution from one of two wildtype alleles strongly impaired p53 system functionality and increased resistance to melphalan. Second hits abolished remaining p53 activity and increased resistance to genotoxic drugs even further. These results fit well with the clinical drive to *TP53* single- and double-hit disease in MM patients, provide a rationale for the most commonly observed double-hit constellation (del17p+ *TP53* point mutation), and underscore the potential increases in MM cell malignancy associated with any type of initial *TP53* lesion.

## Introduction

The past 15 years have seen rapid and ongoing changes in the treatment of multiple myeloma (MM), a plasma cell neoplasm which accounts for about 13% of hematologic cancers^1,2^. These advances have already led to substantial improvements in the numbers for progression-free and overall survival, with ever more therapeutic options available for combinatorial use and promising efficacy even in relapse settings^3^. About 15% of newly-diagnosed MM is classified as “high-risk disease” and expected to show an unfavourable course of treatment with (much) shorter than average survival. This classification is based on retrospective correlation of cytogenetic and clinical parameters with outcome, and is being updated and refined as more molecular information becomes available^4–6^. One of the most constant features in high-risk myeloma are lesions that affect the *TP53* gene, which encodes the tumor suppressor protein p53. Typically, such hits have been associated with deletions of the short arm of chromosome 17 (del17p) and determined cytogenetically by fluorescence *in situ* hybridization^7^. The possibility of involvement of genes other than *TP53* in del17p-associated oncogenicity has still not fully been discounted^8^, but presence of *TP53* in the minimally deleted region and the demonstration that *TP53* hemizygosity entails p53 haploinsufficiency strongly support its cardinal role^8,9^. Additionally, advances in tumor cell sequencing have revealed the existence of point mutations affecting the p53 coding sequence in MM patients. Such mutations are most often observed in association with del17p, thus marking out MM cells with hits to both *TP53* alleles (“double-hit disease”), although combinations of wildtype + mutant can also be found^10–13^. The *TP53* double-hit constellation represents one of the most dire prognosis groups in newly diagnosed MM, whereas hardly any^4^ or only a moderate effect^14^ have been reported for *TP53* single-lesion disease.

Although MM with *TP53* lesions appears to be per se responsive to therapies with novel agents^15–17^, the acquisition of additional oncogenic driver events, often in combination with outgrowth of a *TP53* double-hit clone, appears to underlie the fast progress into intractable and fatal disease^18^. However, little is known about whether and to what extent the different types and constellations of *TP53* lesions, i.e. a deletion-first (haploinsufficiency) vs. a mutation-first (potential dominant negativity) vs. a double-hit scenario (most often high expression of just mutant p53 protein) may affect p53 system functionality and drug responsiveness in MM, and if such knowledge could inform therapeutic decisions. Additionally, depending on the actual mutation present, gain-of-function activities of p53 are also a possibility^19^. Here we have used the *TP53*^wt/wt^ MM cell line AMO-1 in a functional approach that emulates the types of single-hit and double-hit lesions to *TP53* observed in MM patients, and which provides a means to compare their effect within the frame of an isogenic cell line model.

## Results

### Generation of mono-and bi-allelic *TP53* lesions in MM cells and analysis of p53 system functionality in AMO-1 clones

**Figure 1:**
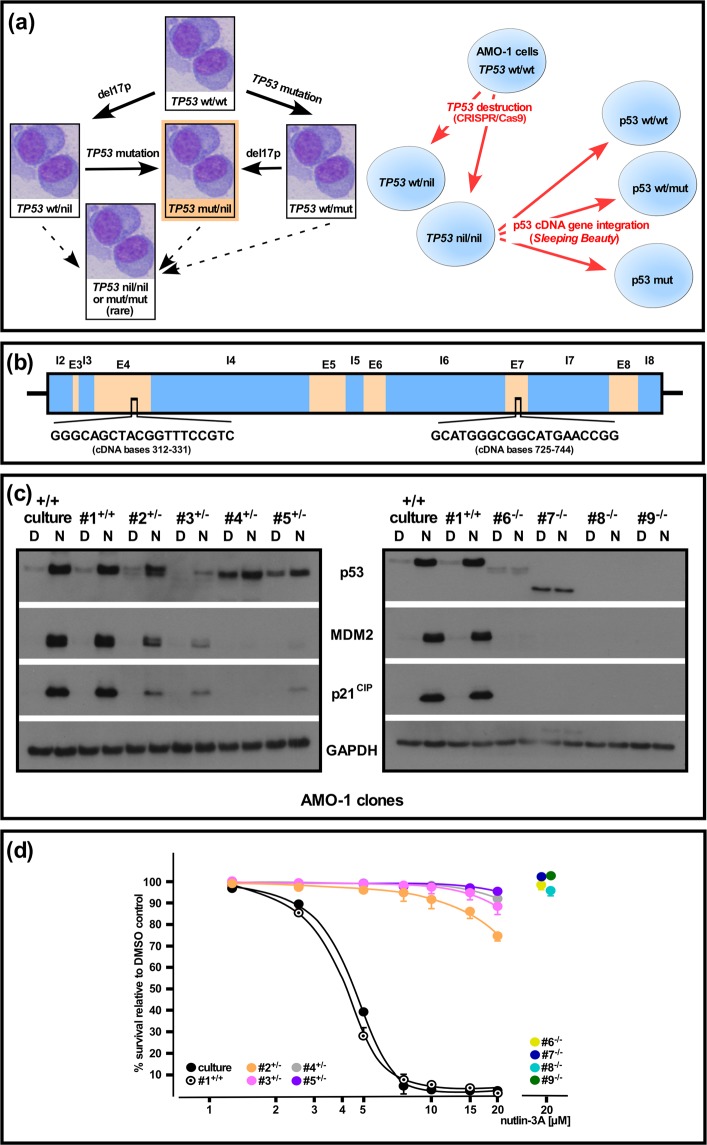
Generation and functional analysis of homo- and heterozygous *TP53* lesions in an AMO-1 MM cell line model system. (**a**) Pathways to *TP53* lesions. Left: Primary MM cells will either initially lose part of chromosome 17 (del17p) or acquire an inactivating *TP53* point mutation as “first-hit”. Both will then normally give rise to a del17p/point mutation “double hit” constellation (highlighted in orange), which is also the most common arrangement found in MM cell lines. Right: *TP53* destruction in AMO-1 cells by CRISPR/Cas9 followed by establishment of p53 cDNA gene expression in *TP53* nil/nil clones via *Sleeping Beauty* to generate p53 wt/wt, p53 wt/mut and p53 nil/mut scenarios. (**b**) Schematic intron (I)/exon (E) structure of parts of the human *TP53* gene, indicating the two target sites for CRISPR/Cas9-mediated disruption. (**c**) Characterization of p53 system functionality in p53 wt AMO-1 cells (culture, clone #1), AMO-1 clones with a single disrupted *TP53* allele (clones 2–5) or with two disrupted *TP53* alleles (clones 6–9). Cells were treated overnight with 10 µM nutlin-3A (N) or the applicable amount of solvent (DMSO, D) and analyzed for p53 system components by Western blotting. Staining for GAPDH served as loading control. See Supplementary Fig. 4 for full representation of the data. (**d**) Kill curves for 3-day-treatment with nutlin-3A for *TP53*^+/+^, *TP53*^+/−^ and *TP53*^−/−^ AMO-1 clones. Only the highest drug concentration tested is depicted for the fully-resistant *TP53*^−/−^ clones (#6–9). Kill curves were calculated from 2 independent experiments. Error bars depict s.e.m.

In order to get better insights into the functional consequences of the different types of *TP53* lesions in MM we decided to try to emulate the different single- and double-hit constellations within a single MM cell line model. The two basic steps involved were an initial destruction of one or both *TP53* alleles in *TP53*-wildtype MM cells via the CRISPR/Cas9 method, followed by concerted introduction of cDNA-expression cassettes for wildtype + mutant p53 via the *Sleeping Beauty* transposon system (Fig. 1a). Two different target sequences for CRISPR/Cas9-mediated *TP53* disruption were tested (Fig. 1b) and the respective guide-RNA expression vectors were co-electroporated with an expression plasmid for EGFP to permit manual selection of the most efficiently transfected cells for further clonal upgrowth. Of the four *TP53* wildtype MM cell lines initially tested (AMO-1, MM.1s, MOLP-8, NCI-H929) only AMO-1 yielded sufficient numbers of clones to permit further analysis, probably due to its favourable combination of relatively high electroporation efficiency for plasmids and fast growth rate. A total of 85 clones were checked for *TP53* defects by PCR/sequence analysis off genomic DNA covering the respective CRISPR/Cas9 target sites and/or Western blotting for p53 and its downstream targets MDM2 and p21^CIP^ after overnight treatment with the MDM2 inhibitor nutlin 3A (Fig. 1c). Clones showing *TP53* defects on sequencing (i.e. a clean sequence turning to unreadability once the sequences between the two alleles diverge) were further characterized by cloning of PCR products into vector pGEM-T Easy to assess whether one or both *TP53* alleles were affected and to identify the precise molecular defects. One *TP53*^wt/wt^ clone (clone #1) and four clones each with either *TP53*^wt/−^ or *TP53*^−/−^ status were selected for further analysis (Fig. 1c,d and See Supplementary Fig. 1 for an overview of the specific changes to *TP53* and the predicted effects on the translated p53 protein). As expected, the wildtype clone was indistinguishable in its reactions to treatment with nutlin 3A from the parental cell line, whereas clones with double disruption of *TP53* showed no activity of the p53 system at all, as evidenced by absence of upregulation of p53/MDM2/p21^CIP^ in Western blotting and of nutlin 3a-induced cell death (Fig. 1c,d). Clones “hemizygous” for *TP53* (in the sense that they harbored one wildtype allele and one affected by a CRISPR/Cas9-mediated indel with presumed lack of contribution to p53 levels from the affected allele) displayed some variability in their ability to mount a nutlin-induced p53 response, but these effects were in all cases strongly impaired compared to parental AMO-1 cells with two wildtype copies of *TP53*, and barely affected their survival when challenged with the MDM2 inhibitor (Fig. 1c,d).

### Functional analysis of the effect of p53 point mutation in the AMO-1 MM cell line model system

**Figure 2:**
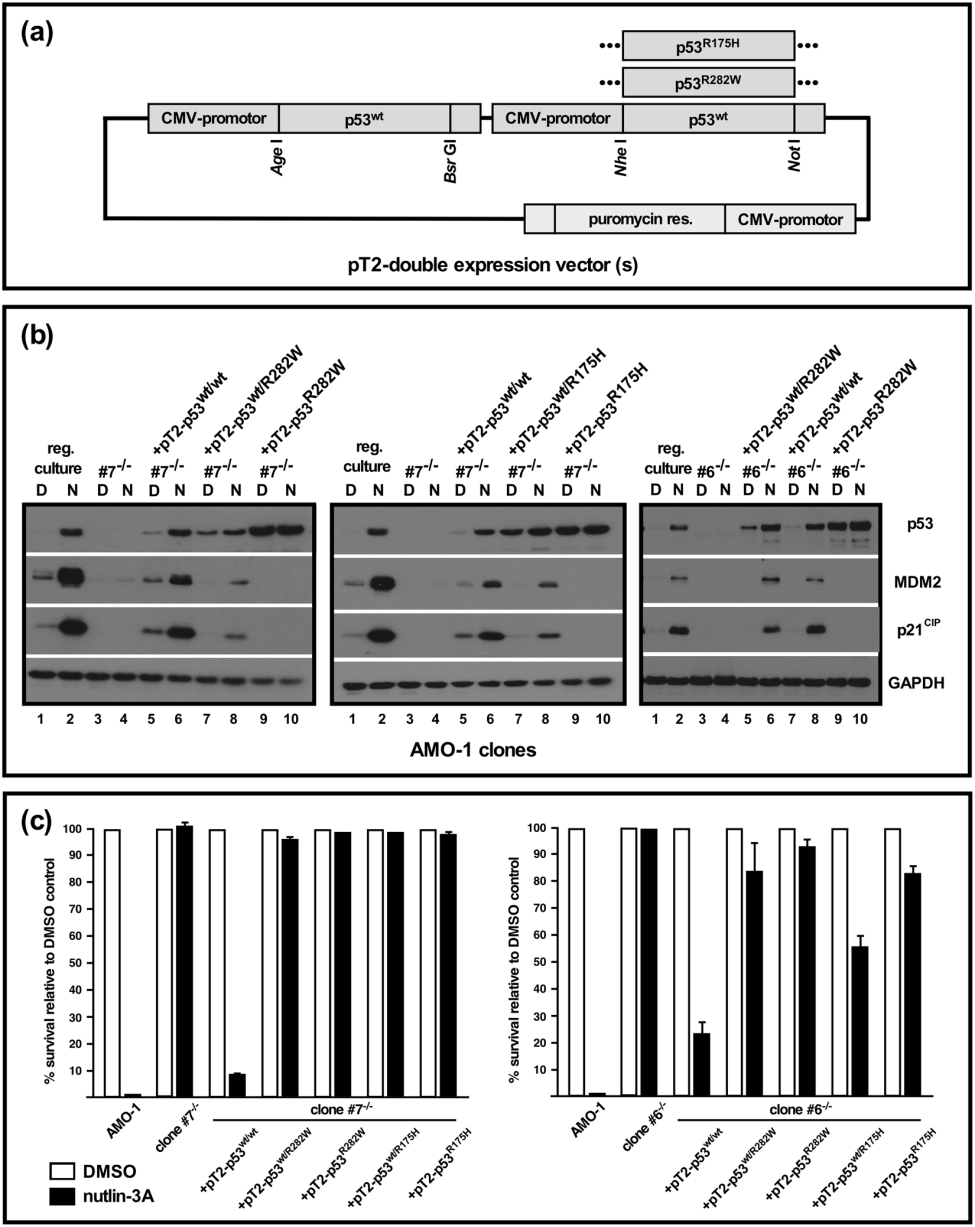
Functional analysis of single p53 point mutations with or without concomitant expression of wildtype p53 in AMO1-*TP53*^−/−^ cells. (**a**) Schematic representation of *Sleeping Beauty* vectors with two individually addressable CMV promotor-driven expression cassettes to establish stably transfected polyclonal cultures with p53^wt/wt^, p53^wt/R282W^ or p53^wt/R175H^ cDNA gene expression. (**b**) *Sleeping Beauty*-mediated introduction into AMO-1 *TP53*^−/−^ cells (clone #7) of either two CMV promotor-driven cDNA genes for wildtype p53 (pT2-p53^wt/wt^), of one wildtype plus one mutant cDNA gene (pT2-p53^wt/R282W^ (left, in clone #7; right, in clone #6)) or pT2-p53^wt/R175H^ (middle, in clone #7), or of single mutant p53 cDNA genes, to emulate “mutation first” and “deletion plus mutation” scenarios. Western blotting for p53 system components after overnight challenge with 10 µM nutlin-3A. See Supplementary Fig. 5 for full representation of the data. (**c**) Survival analysis (annexin V/PI-staining) for two AMO-1 *TP53*^−/−^ clones expressing different constellations of wildtype and mutant p53 after 3 days incubation with nutlin-3A or an applicable amount of solvent (DMSO). Kill rates calculated from 2 experiments. Error bars depict s.e.m.

We next tested whether stable integration of vectors for constitutive re-expression of p53 (i.e. of cDNA copies) into AMO-1 *TP53*^−/−^ clones was feasible and sufficient to re-instate a functional p53 system. A modified *Sleeping Beauty* transposon vector (Fig. 2a and Supplementary Fig. 2) was used to concomitantly introduce either two identical CMV-promotor driven expression cassettes for wildtype p53, or for combinations of wildtype p53 plus point mutated p53, thus emulating a “mutation first” scenario. Two different point mutants, corresponding to known mutation hotspots (R175H and R282W) were used and transposed into *TP53*^−/−^ clones. Initial transient transfection experiments and reverse transcription cDNA analysis with p53/vector-specific primers confirmed that both alleles were about equally well expressed from such double-cassette vectors (Supplementary Fig. 2). Following electroporation and puromycin selection transposed cultures were ready for use in experiments within 12–14 days. The polyclonal cultures that resulted for *TP53*^−/−^/p53^wt/wt^ cells showed only slightly elevated steady state levels of p53 protein when compared to regular AMO-1 cells by Western blotting (Fig. 2b: left and middle: lanes 5 vs. lanes 1, right: lane 7 vs. lane 1). Levels of p53 were strongly enhanced after overnight treatment with nutlin-3A and roughly on a par with the increase observed in parental *TP53* wildtype cells (Fig. 2b: left and middle: lanes 6 vs. lanes 2, right: lane 8 vs. lane 2), showing that the introduction of two constitutively expressed wildtype p53 cDNA-gene copies under CMV-promotor control could still functionally be integrated into the MDM2/p53 degradation/transcription cycle that governs the levels of p53 in AMO-1 cells. Likewise, MDM2 inhibitor treatment led to increased levels of the p53 downstream targets MDM2 and p21^CIP^, although not necessarily to levels as high as in parental AMO-1 cells (Fig. 2b). However, functional reinstatement of the p53 system in knockout clones also largely restored the p53-mediated cell death response (Fig. 2c), showing that this cellular model system indeed displayed all basic features required to analyze p53 function. We therefore tested next the stable transposition of *TP53*^−/−^ cells with *Sleeping Beauty* vectors expressing combinations of wildtype p53 and either the mutant R282W (Fig. 2b, left, clone #7; right, clone #6) or the mutant R175H (Fig. 2b, clone 7, middle). In addition to higher steady-state levels of p53 (Fig. 2b: left and middle: lanes 7 vs. lanes 1; right: lane 5 vs. lane 1) these cells also showed attenuated nutlin-3A-mediated p53 responses with modest increases in total levels of p53 and smallish increases in the levels of MDM2 and p21^CIP^ (Fig. 2b). Accordingly, these changes were not sufficient to trigger substantial cell death responses (Fig. 2c), underpinning the dominant negative functional effect that these p53 mutants are supposed to exert on the tetrameric transcription factor. As expected, introduction of just a single mutant p53 cDNA gene led to high steady-state levels of p53 that remained unchanged by treatment with nutlin-3A (Fig. 2b: lanes 9,10), and no functional responses of the p53 system (Fig. 2b,c) – a situation mimicking that encountered in many MM cell lines which display a *TP53*^−/mut^ phenotype^20^.

### Impact of *TP53*/p53 status on the sensitivity for genotoxic drugs and proteasome inhibitors in the AMO-1 MM cell line model system

**Figure 3:**
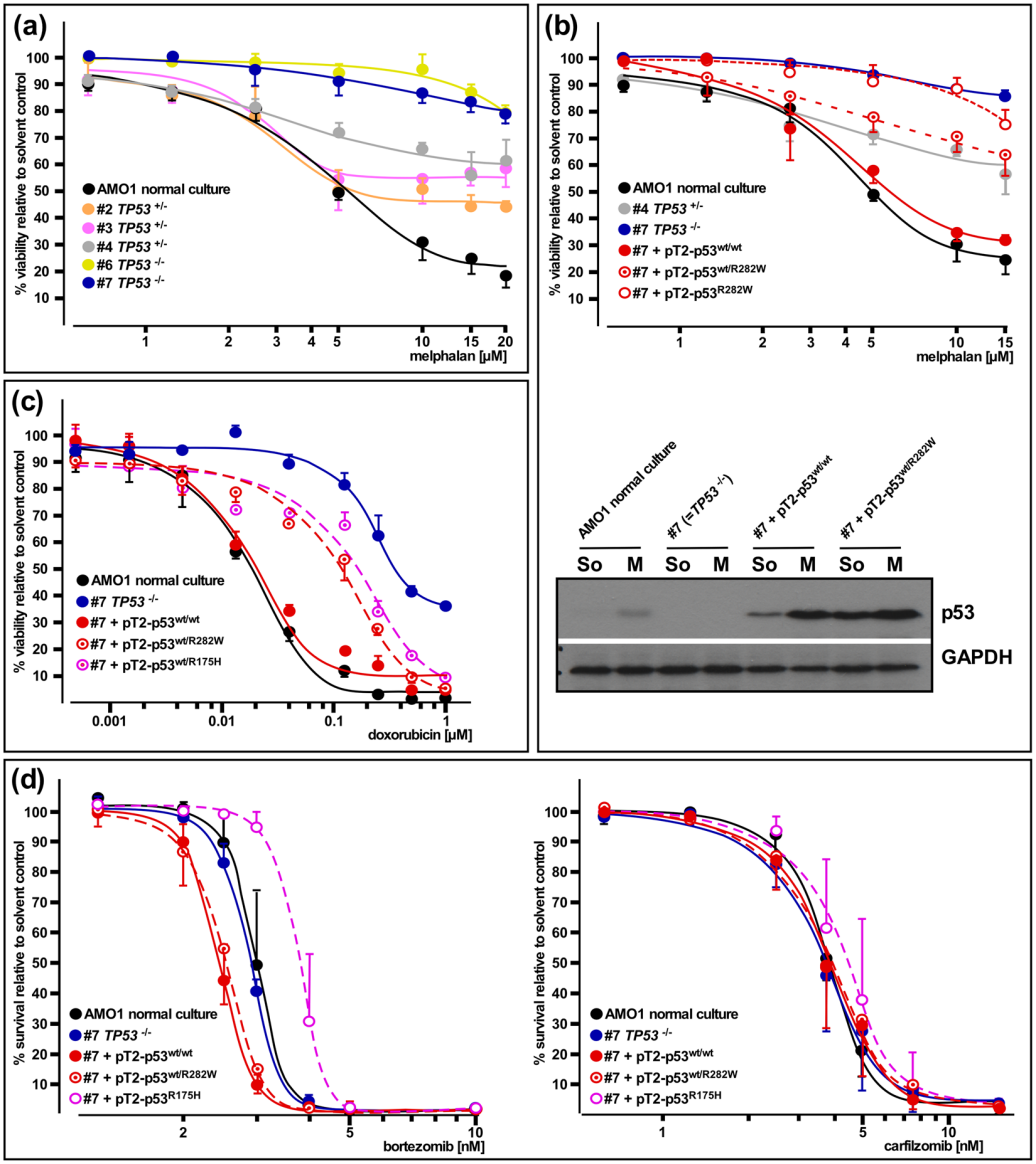
Analysis of drug efficacy as a function of the *TP53*/p53 status. (**a**) Response to melphalan in AMO-1 wildtype cells and various clones with either one or both *TP53* alleles disrupted. Viability assays (alamarBlue) after 3-day drug treatment. (**b**) Top: Response to melphalan in AMO-1 *TP53*^−/−^ cells without (blue curve) or with (red curves) stable expression of combinations of wildtype and mutant p53 cDNA genes via *Sleeping Beauty*. Wildtype (black curve) and *TP53* hemizygous AMO-1 clone #4 (grey curve) also included for comparison. Viability assays (alamarBlue) after 3-day drug treatment. Bottom: Western blot showing melphalan treatment-induced increases in p53 in AMO-1 cells with at least one (cDNA-) gene for p53. See Supplementary Fig. 5 for full representation of the data. (**c**) Response to doxorubicin in AMO-1 *TP53*^−/−^ cells without (blue curve) or with (reddish curves) stable expression of combinations of wildtype and mutant p53 cDNA genes via *Sleeping Beauty*. Two different p53 mutant proteins (R175H, R282W) tested. Wildtype (black curve) AMO-1 cells also included for comparison. Viability assays (alamarBlue) after 3-day drug treatment. (**d**) Response to proteasome inhibitors in AMO-1 *TP53*^−/−^ cells without (blue curve) or with (reddish curves) stable expression of combinations of wildtype and/or mutant p53 cDNA genes via *Sleeping Beauty*. Wildtype (black curve) AMO-1 cells also included for comparison. Survival assays (annexin V/propidiumiodide) after 3-day drug treatment. Kill curves calculated from 3 experiments. Error bars depict s.e.m.

We next tested the consequences of *TP53* single or double deletion for the sensitivity of AMO-1 cells against the clinically employed alkylans melphalan. AMO-1 clones that had either one or both alleles of *TP53* destroyed by CRISPR/Cas9-mediated disruption were treated with the drug and viability curves (alamarBlue metabolic assay) measured after three days (Fig. 3a). The effect of the drug at higher concentrations was severely blunted by hemizygosity for *TP53* (Fig. 3a: clones #2, #3, #4), and became very small in *TP53*^−/−^ clones (Fig. 3a: clones #6, #7). We then tested the effects of melphalan treatment on AMO1 *TP53*^−/−^ cells (Fig. 3b: clone #7, Supplementary Fig. 3a: clone #6, 3b: clone #7) after *Sleeping Beauty*-mediated stable introduction of two cDNA genes for either wildtype p53 or for combinations of wildtype and mutant p53 (Fig. 3b and Supplementary Fig. 3). Whereas re-instatement of a wildtype p53 status led to near-complete resensitization against melphalan, expression of the wildtype + mutant combinations produced an attenuated response very similar in size to that seen in the CRISPR/Cas9-generated clones hemizygous for *TP53*, again underpinning a dominant negative effect of acquisition of a *TP53* mutation in a “mutation first” scenario (Fig. 3b and Supplementary Fig. 3). Accordingly, when comparing the effects of melphalan treatment on the p53 levels in *TP53*^−/−^ cells either transposed with the p53^wt/wt^ or the p53^wt/R282W^ combination, the latter showed a decidedly smaller relative increase in p53 levels (Fig. 3b; absolute levels are higher in these cells because of the different steady-state level due to the presence of mutant p53, see also Fig. 2b). As expected, expression of mutant p53 in the *TP53*^−/−^ cells did not alter the already unresponsive behaviour of such clones in any way (Fig. 3b). Treatment of AMO-1 cells with doxorubicin, another genotoxic compound that exerts at least part of its anti-myeloma effects via the p53 pathway, also showed a strong influence of the p53 status (Fig. 3c). Again, both settings emulating a “mutation-first” scenario (introduction of p53^wt/R175H^ or p53^wt/R272W^ into *TP53*^−/−^ cells) led to levels of resistance decidedly higher than normal AMO-1 *TP53* wildtype cells, or those in which two copies of p53 wildtype cDNA genes were expressed, but did not quite attain the insensitivity imparted by full p53 loss (Fig. 3c). Lastly, we tested the influence of the p53 status on the sensitivity of AMO-1 cells for proteasome inhibitors. No differences in the reaction to carfilzomib was found for any of the different *TP53*/p53 constellations (Fig. 3d, right), and at best slight shifts of the kill curves were found with bortezomib treatment. These were, if significant at all, perhaps a reflection of the slightly higher levels of wildtype p53 protein in the CMV-promotor driven contexts (resulting in slight sensibilisation) and of strong overexpression of mutant p53 in the mutant only expression scenario (resulting in slight desensibilisation). No difference was found between AMO1 wildtype cells and the *TP53*^−/−^ clone (Fig. 3d, left), confirming that functionally p53 appears not to be significantly involved in the proteasome inhibitor-mediated apoptosis of these cells.

## Discussion

The presence of genetic lesions affecting both alleles of the tumor suppressor gene *TP53*, most commonly achieved via a combination of deletion (del17p) and point mutation, has now firmly been established as the major indicator for unfavourable prognosis in newly diagnosed multiple myeloma^4,14,21^. Additionally, a sizeable subgroup of MM patients will acquire *de novo* first hits to *TP53*, and/or progresses from single-hit to double-hit constellations during the course of their disease under treatment, thus in effect genetically switching into a class with high-risk features^11–13,22,23^. These observations also suggest that, whereas clones that harbour a single hit to *TP53* do have a selection advantage, acquisition of a second hit still provides a significant, and probably often decisive step towards intractable malignancy. However, whereas it is now possible to monitor the presence, emergence or rise of subclones with *TP53* deficiencies at consecutive stages of the disease, it remains unclear if, when and how such knowledge could potentially be used to inform therapeutic decisions.

Here, we have for the first time approximated the main types of *TP53* lesion observed in MM patients within the frame of a single isogenic MM cell line model, and analyzed their impact on the functionality of the p53 system and their potential role for resistance against anti-MM drugs. Importantly, these models also comprise the “single-hit” constellations, i.e. situations in which either p53 contribution from one of two *TP53* alleles is lost (approximating the del17p case), or in which only one of two expressed *TP53* alleles is point mutated. The first situation has previously been associated with haploinsufficiency^10^, whereas the second situation (normally associated with dominant negativity) is encountered in MM patients, but is not represented by any MM cell line.

With a number of different haploinsufficient clones and two different p53 hotspot mutations tested, both types of single-hit models strongly impaired the functionality of the p53 system, and attenuated the response of AMO-1 cells to genotoxic drugs to similar extent. Furthermore, acquisition of a second hit to *TP53* (here approximated by different models that either lead to strong expression of only mutant p53 protein or no expression of any p53 protein at all) again produced identical effects inasmuch as even the last vestiges of p53 system functionality were abolished and resistance to genotoxic drugs was still further enhanced. These results are important for three reasons: First, these models functionally underscore the strong gains in fitness and drug resistance that MM cells can potentially acquire from any type of single *TP53* lesion, and which supports their rise to subclonal prominence. Second, the observation that the second hit to *TP53* still further increases the survival potential of single-hit AMO-1 cells corresponds well with the clinically observed drive towards “double-hit” disease. Full *TP53* inactivation in combination with genetic events that boost proliferation probably provides one of the fastest routes to outgrowth of intractable MM subclones. Third, these data lend support for the notion that it is indeed the hit to *TP53* – and possibly to *TP53* only – that represents the oncogenic element in the del17p lesion, and that it is the associated loss of p53 system function (and not any gain-of-function mechanism) that generally drives the spread of *TP53* lesions across the MM holotumor. If, as implied by our experiments, double-hit disease is the favoured outcome, and in addition the functional consequences of single *TP53* deletion and single *TP53* mutation are essentially the same, then the selection of the generally observed constellation of bi-allelic *TP53* lesions: del17p+ mutation, makes perfect sense. If a deletion is the easiest way to lose a functional *TP53* copy, but a second deletion poses a problem because it normally also entails bi-allelic loss of other genes that might be important for MM cell fitness (but which are not necessarily related to malignancy), then the second event is most likely a precise hit to *TP53*, i.e. an inactivating mutation. Conversely, if that precise hit happened to be the initial lesion, it should most often be followed by del17p, providing an MM cell with all the gains of removing the remaining wildtype *TP53* allele at no or little collateral costs.

Even though no single cell line model is likely to reflect all aspects of the consequences of *TP53* lesions in MM patients, the AMO-1 system represented here has proven a robust and coherent model system for functional and pharmacological analysis of the consequences of single- and double-hits to *TP53*. Importantly, it implies that the different types of single-hit clones may already acquire considerable resilience against genotoxic drugs, but that this does not represent a primary resistance mechanism against other therapeutic approaches, such as proteasome inhibitors. The clinical need for treatment of *TP53* double-hit MM patients is imminent, but unfortunately there are no proven drug approaches that target this lesion, as efforts to pharmacologically re-activate mutant p53 have not yet led to specific therapies^24^. Moreover, since MM cells are adept at developing resistance also against therapies that do not rely on functional p53, it is possible that even with the best efforts, and including the use of novel approaches such as immunotherapies, MM patients with double-hits to *TP53* may always represent the clinically most disfavoured group. From a longer-term perspective it may therefore be rewarding to identify therapy approaches with the declared aim to keep the number of MM patients that develop *TP53* double-hit disease as low as feasible. Increasingly precise molecular tumor diagnostics, including MRD molecular status assessments, can now be applied before and along the course of treatment. The presence or rise of established or novel MM subclones with single or double *TP53* deficiencies could thus be detected and the information potentially be used for therapeutic decisions as well as for retrospective analyses that promote our understanding of the precise genetic features that underlie the unfavourable clinical courses of MM with *TP53* lesions. Based on the results presented here, it would for example be important to know if patients who present with MM subclones that display single hits to *TP53* should rather not be treated with genotoxic drugs in order to minimize the risk of acquisition of the second hit. Likewise, it would be important to know if patients with MM subclones that display single *TP53* lesions should preferentially be treated with therapies that are known to work on MM cells with *TP53* defects, and if such genetic constellations are an indication for use of powerful combination approaches, perhaps with inclusion of immunotherapies, in an attempt to nip emergent (pre-) high risk clones in the bud. Over time, such information will automatically accrue as part of the collective treatment histories of the global MM patient population, but it will only be interpretable if all aspects regarding the (change of) status of *TP53* lesion are precisely documented.

## Materials and Methods

### Cell culture

Cell line AMO-1 was bought from the German Collection of Microorganisms and Cell Cultures (DSMZ, Braunschweig, Germany) and immediately expanded into stock and working banks. The cell culture was reinstated from working bank-aliquots every 3–4 months. Standard MM cell culture was performed in RPMI-1640 medium supplemented with 10% FBS, 1 mM sodium pyruvate, 2 mM glutamine, 100 U/ml penicillin, and 100 µg/ml streptomycin. Cells were kept at 5% CO_2_ and 37 °C. Cultures were regularly checked for mycoplasma negativity^25^.

### CRISPR/Cas9 constructs

Vectors for CRISPR/Cas9-mediated *TP53* disruption were based on the GeneArt CRISPR nuclease: CD4 enrichment kit (Invitrogen, Carlsbad, USA; A21175). The following oligonucleotides were used to create dsDNA fragments for cloning and generation of functional guide-RNA expression vectors (nucleotide sequence corresponding to the human *TP53* gene in bold): construct 1: TP53-1-forward: 5′-**GGGCAGCTACGGTTTCCGTC**GTTTT-3′, TP53-1-reverse: 5′-**GACGGAAACCGTAGCTGCCC**CGGTG-3′; construct 2: TP53-2-forward: 5′-**GCATGGGCGGCATGAACCGG**GTTTT-3′, TP53-2-reverse: 5′ **CCGGTTCATGCCGCCCATGC**CGGTG-3′.

### Determination of the *TP53* status of MM cell line clones

Clones were initially screened for p53 system functionality while still in 96-well format culture, by exposing about 10.000 cells overnight to either DMSO or 10 µM nutlin-3A, followed by Western analysis for p53 system induction. Additionally, genomic DNA was prepared for all clones and parts of the *TP53* gene which included the CRISPR target site(s) amplified by conventional PCR using primers 5′-TGAAGACCCAGGTCCAGATG and 5′-CACTGACAGGAAGCCTAAGG (site 1) and/or 5′-TTGCCACAGGTCTCCCCAAG and 5′-TGAGTGGGAGCAGTAAGGAG (site 2), respectively. PCR products were Sanger sequenced to either confirm wildtype status (clean correct reads) or presence of single/double lesions (clean reads turning unreadable from the base at which both alleles diverge, or in one case (clone #8) a clean read containing an insertion). The latter clones were further characterized by cloning of PCR products into vector pGEM-T Easy and Sanger sequencing of minipreparations to (i) reveal the exact lesion(s) present, (ii) confirm the presence of wild-type *TP53* in single-lesion clones (Supplementary Fig. 1).

### Construction of p53 cDNA expression vectors

Wild-type p53 cDNA was amplified from AMO-1 first-strand cDNA using primers that flank the coding region either with *Nhe*I/*Not*I or with *Bsr*GI/*Age*I restriction sites and cloned into suitably modified pBluescript II vectors. Single base changes to generate p53 hotspot mutants R175H (selected for its presence and previous confirmation of p53 system loss of function in MM cell line OPM2^20^) and R282W (selected for its presence in a primary MM sample showing additional loss of the initially remaining wildtype *TP53* gene in a later biopsy) were introduced according to the QuikChange protocol. A pT2 (*Sleeping Beauty*) transposon vector conferring puromycin resistance was modified to contain either a single or two near identical CMV promotor-driven expression cassettes. In the latter case, the only difference between these cassettes was that they were individually selectable for subcloning using either the *Nhe*I/*Not*I or the *Bsr*GI/*Age*I combinations. Wildtype and/or mutant p53 cDNAs were finally subcloned from the respective pBluescript II vectors into *Sleeping Beauty* to generate vectors for p53^wt/wt^ and p53^wt/mut^ cDNA-gene expression, or for expression of single p53 cDNA-gene copies.

### Electroporation and selection of AMO-1 clones or transposed cultures

The protocol for MM cell electroporation is described in detail in ref. ^26^. *TP53* guide-RNA expression vectors were used in electroporation mixtures at concentrations of 25 µg/ml when used alone, or at 20 µg/ml each when used together. Additionally, an expression plasmid for EGFP was co-transfected, and after 3 days single bright green cells were manually selected from the transfection culture and transferred into 96-well plates for clonal upgrowth and characterization of *TP53* defects.

Electroporation of *Sleeping Beauty* vectors into AMO-1 cells was performed with 20 µg/ml of *Sleeping Beauty* plasmid, 30 µg/ml of pTX100 transposase expression plasmid and 5 µg/ml of EGFP expression plasmid for easy assessment of transfection efficiency. After overnight recovery the transfected cultures were subjected to puromycin selection (1 µg/ml, 10–14 days) to establish polyclonal cultures of transposed cells.

### Drug treatment of MM cells

MM cells were seeded into 96 well plates (10.000 cells per well), incubated for 3 days with the respective compounds and subjected to alamarBlue or annexin V/propidiumiodide viability/cell death measurements. Drug solutions were always freshly prepared from concentrated stocks (solvents for stock solutions: acidic ethanol for melphalan, DMSO for all other compounds). Drug treatment of cells intended for use in Western blotting was performed overnight.

### Western blotting and antibodies

Western blotting was performed exactly as described^27^. The primary antibodies used were: anti-β-actin (Sigma, Deisenhofen, Germany; A5316), anti-MDM2 (Santa Cruz, Heidelberg, Germany (sc-965)), anti-p21^Cip/Waf^ (Santa Cruz; sc-397), anti-p53 (Santa Cruz; sc-126), and anti-GAPDH (Sigma; G9545). Secondary antibody F(ab′)2 fragments coupled to horseradish peroxidase and specific for rabbit-IgG (No. 111-036-045) or mouse-IgG (No. 115-036-072) were obtained from Jackson ImmunoResearch, Newmarket, UK. A freshly made solution of luminol (2.5 mM), p-coumaric acid (0.2 mM) and H_2_O_2_ (0.01%) in 100 mM Tris-HCl (pH 8.8) was used as reagent for chemiluminescent detection.

### Other reagents

Annexin V was prepared following a published protocol^28^, and coupled to either fluoresceinisothiocyanate (Sigma; F7250) or to PromoFluor 647 using its N-hydroxysuccinimidyl ester (PromoCell, Heidelberg, Germany; PK-PF647-1). Drugs used in this study were obtained from Sigma-Aldrich (melphalan; M2011), Selleck Chemicals, München, Germany (bortezomib; S1013, carfilzomib; S2853, nutlin-3A; S8059), and Merck, Darmstadt, Germany (doxorubicin; 324380). A solution of Na-resazurin (Sigma; R7017) in PBS was used as reagent in viability assays (alamarBlue).

### Flow cytometry

Cells were washed with PBS, pelleted and resuspended in 200 µl of cold annexin V binding buffer (10 mM HEPES/NaOH, 140 mM NaCl, 2.5 mM CaCl_2_; pH7.4) containing 1 µl of annexin V-PromoFluor 647 solution (see Reagents) and 1 µg/ml propidium iodide. Flow cytometry was performed with a FACS Calibur (BD Biosciences, Heidelberg, Germany). Datafiles were analysed with FlowJo version 8.8.7 (Tree Star, Inc., Ashland, USA).

### Data analysis

Dose-effect curves were calculated from at least two independent experiments by non-linear regression analysis (sigmoidal shape, variable slope setting) using GraphPad Prism 7 (GraphPad Software, La Jolla, CA, USA). Regression analysis was not performed for data sets when it was obvious that a dose-effect relationship did not exist. The figures depict functional effects calculated with respect to the individual solvent treated controls of the respective clones. Data permitting appraisal of the magnitude of differences (generally slight) between the clones or *Sleeping Beauty*-transposed polyclonal cultures and the parental cell line are presented in Supplementary Figs. 1c and 3c.

All data generated or analysed during this study are included in this published article (and its Supplementary Information Files). The described materials are freely available for scientific purposes.

## Supplementary information

Supplementary Information
