# Supplementary material for: Assessment of TP53 lesions for p53 system functionality and drug resistance in multiple myeloma using an isogenic cell line model
Source: Sci Rep. 2019 Dec 2;9:18062. doi: 10.1038/s41598-019-54407-4 (PMC6889167; doi:10.1038/s41598-019-54407-4)
Supplement: Supplementary file 1 — Supplementary Information [file 41598_2019_54407_MOESM1_ESM.pdf]

Assessment of *TP53* lesions for p53 system functionality and drug resistance in multiple myeloma using an isogenic cell line model

Umair Munawar, Markus Roth, Santiago Barrio, Harald Wajant, Daniela Siegmund, Ralf C. Bargou, Martin Kortüm and Thorsten Stühmer

Supplementary Data

Supplementary Figure 1. (a), (b): Analysis of CRISPR/Cas9-generated *TP53* indels in AMO-1 clones. PCR products amplified off genomic DNA preparations and covering the CRISPR target region were cloned into the vector pGEM-T Easy and plasmid minipreparations from several bacterial colonies made and sequenced. Excerpts from the individual reads are shown to indicate indels present in the respective AMO-1 clones. To the right the predicted protein changes are indicated. (c) left-hand table: Absolute survival data for the solvent-treated controls of AMO-1 clones in comparison to the parental cell line. The experimental numbers given are the percentage of cells that are annexin V- and propidiumiodide-negative. right-hand table: Overview of the differences in viability reads of solvent controls for the AMO-1 clones tested with alamarBlue viability assays. Here, for each single experiment the numbers given indicate the percentage of effect relative to that of the parental cell line (which is therefore depicted as '100').

Supplementary Figure 2. Equal expression system for p53 cDNA genes. Top: Schematic representation of pT2 (*Sleeping Beauty*) vector with two individually addressable CMV-promotor-driven expression cassettes. Bottom: cDNA-analysis showing equal expression of wildtype and mutant (R175H) p53 cDNA-genes from JJN-3 cells transiently transfected with a pT2-p53$^{wt/R175H}$ double cassette vector.

Supplementary Figure 3. (a), (b): Functional analysis of p53 mutation with or without presence of wild-type p53 in *TP53*$^{-/-}$ AMO-1 cells. Same type of experiments as shown in Figure 3, but different CRISPR/Cas9 *TP53* double-hit clone (a) and/or different p53 mutant (R175H) (b) used. Viability assays (alamarBlue) after 3-day drug treatment. (c): left-hand table: Absolute survival data for the solvent-treated controls of AMO-1 clone #7 Sleeping Beauty-transposed variant cultures in comparison to the parental cell line. The experimental numbers given are the percentage of cells that are annexin V- and propidiumiodide-negative. right-hand table: Overview of the differences in viability reads of solvent controls for the AMO-1 clone #7 Sleeping Beauty-transposed variant cultures tested with alamarBlue viability assays. Here, for each single experiment the numbers given indicate the percentage of effect relative to that of the parental cell line (which is therefore depicted as '100').

Supplementary Figure 4. Ponceau Red stains of membranes and uncropped representations of x-ray films used in the assembly of Figure 1b. For Western analyses of p21, p53 and MDM2 the membrane was sectioned into three pieces guided by the bilaterally positioned size markers. Antibody staining for these three proteins was thus always performed on a virginal piece of membrane. GAPDH staining was subsequently performed on the piece of membrane containing the p53 band without an intermediate stripping process.

Supplementary Figure 5. Uncropped representations of x-ray films used in the assembly of Figure 2b. For Western analyses of p21, p53 and MDM2 the membrane was sectioned into three pieces guided by bilaterally positioned size markers (Picture of Ponceau Red stain only available where shown). Antibody staining for these three proteins was thus always performed on a virginal piece of membrane. GAPDH staining was subsequently performed on the 2 re-adjusted pieces of membrane containing the p53 and p21 bands (because it was not immediately clear on which piece the signal would be located) without an intermediate stripping process. The leftmost two bands in the pictures shown here for Figure 2b, right, have been cropped away for representation in actual Figure 2b, because the setting they represent

(Sleeping Beauty transposition of a single p53 wildtype cDNA gene) was not part of the other two experiments shown.

Supplementary Figure 6.

Ponceau Red stain of the membrane and uncropped representations of x-ray films used in the assembly of Figure 3b. GAPDH staining was performed after p53 staining and without an intermediate stripping process.

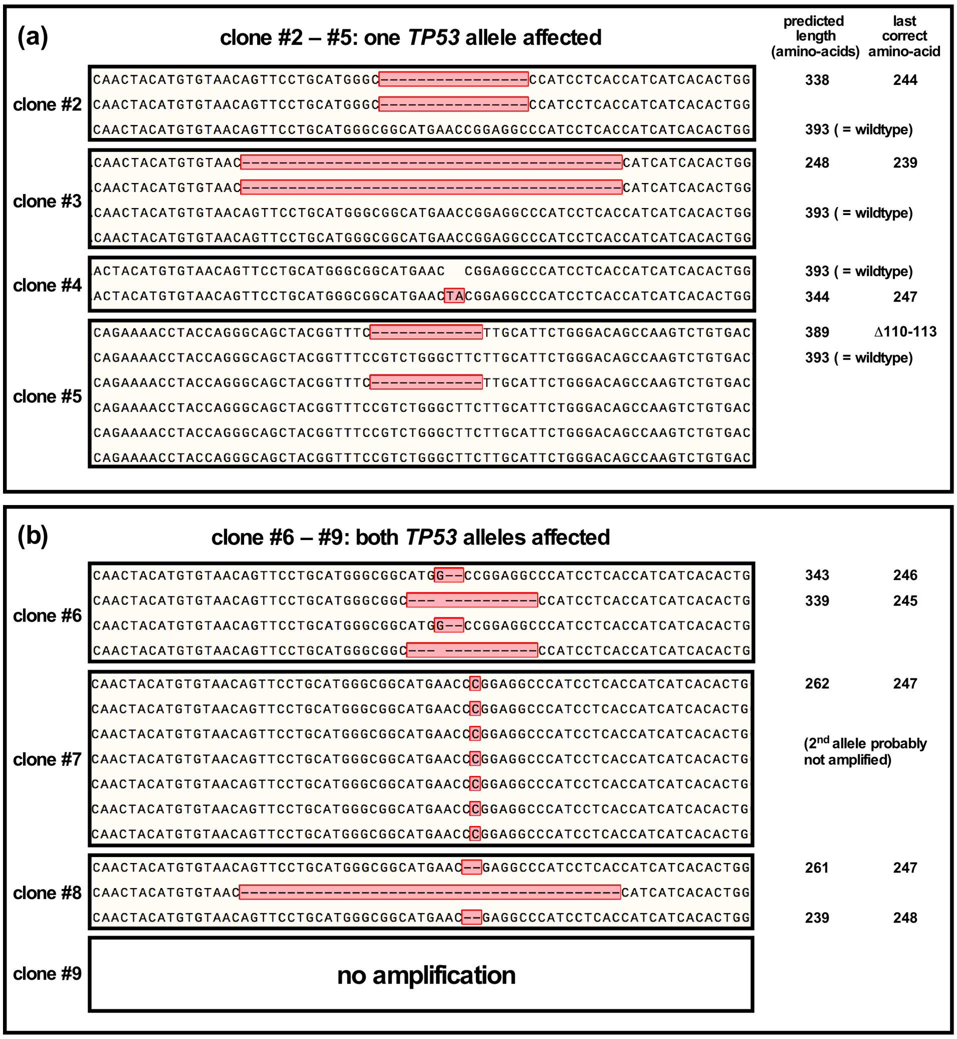

(c)

Annexin V survival assay (Figure 1d)

	Exp.1	Exp.2	Mean	SD	rel. to AMO-1 cell line
AMO-1 cell line	95,5	95,2	95,35	0,21	100
clone #2	93,3	93,7	93,5	0,28	98
clone #3	94,5	93,6	94,05	0,64	99
clone #4	93,7	93,8	93,75	0,07	98
clone #5	94,5	95,1	94,8	0,42	99
clone #6	94,9	95,4	95,15	0,35	100
clone #7	94,4	94,1	94,25	0,21	99
clone #8	95,2	94,9	95,05	0,21	100
clone #9	89,8	89,1	89,45	0,49	94

alamarBlue viability assay (Figure 3a)

	Exp.1	Exp.2	Exp.3	Mean	SD
AMO-1 cell line	‘100’	‘100’	‘100’	‘100’	
clone #2	84,3	96,7	100	93,7	8,25
clone #3	88,6	94,1	97,9	93,5	4,63
clone #4			not tested		
clone #5	83,3	89,3	89,4	87,3	3,47
clone #6	85,6	101,2	106	97,6	10,65
clone #7	90,2	94,4	101,8	95,5	5,87
clone #8			not tested		
clone #9			not tested		

Supplementary Figure 1

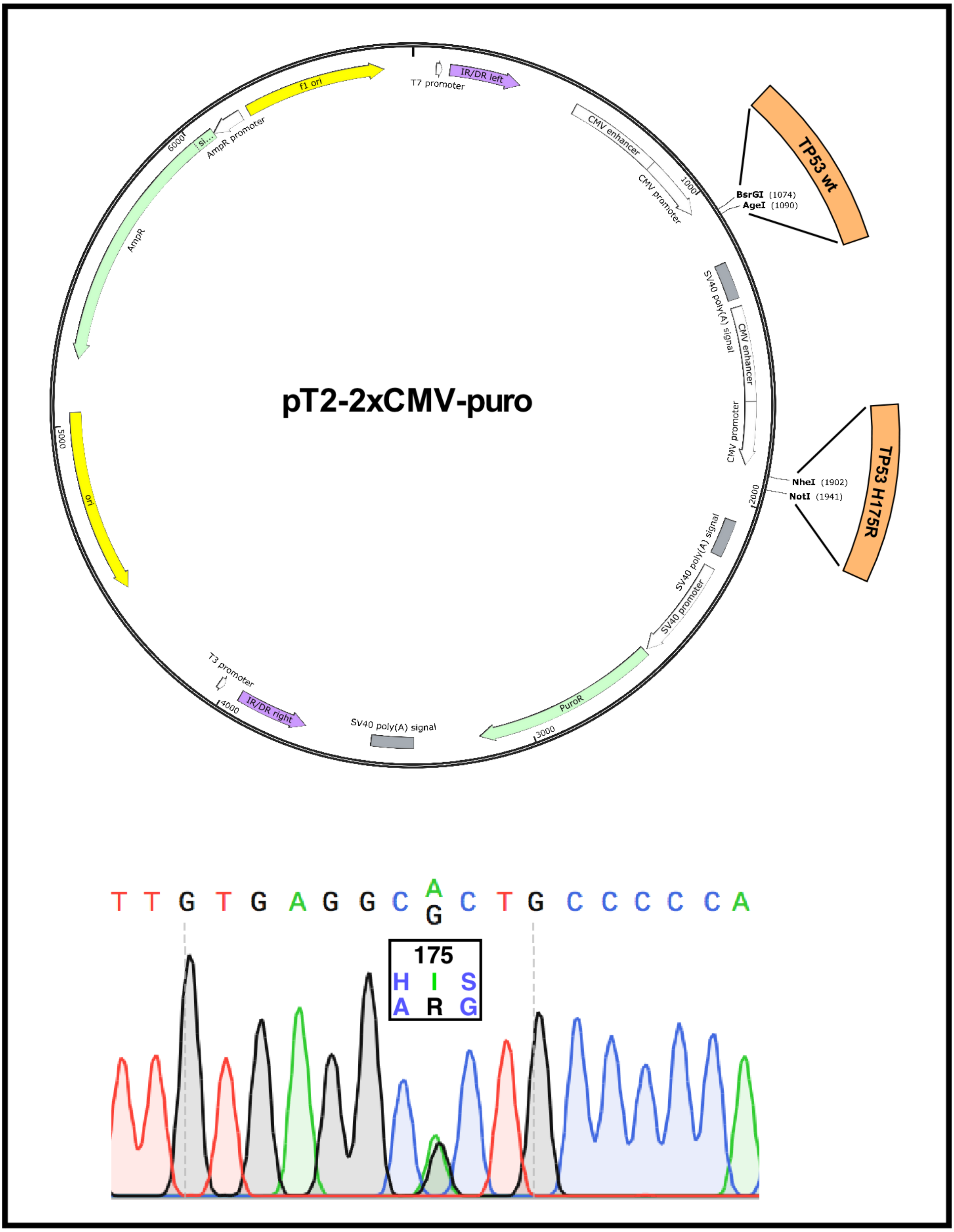

Supplementary Figure 2

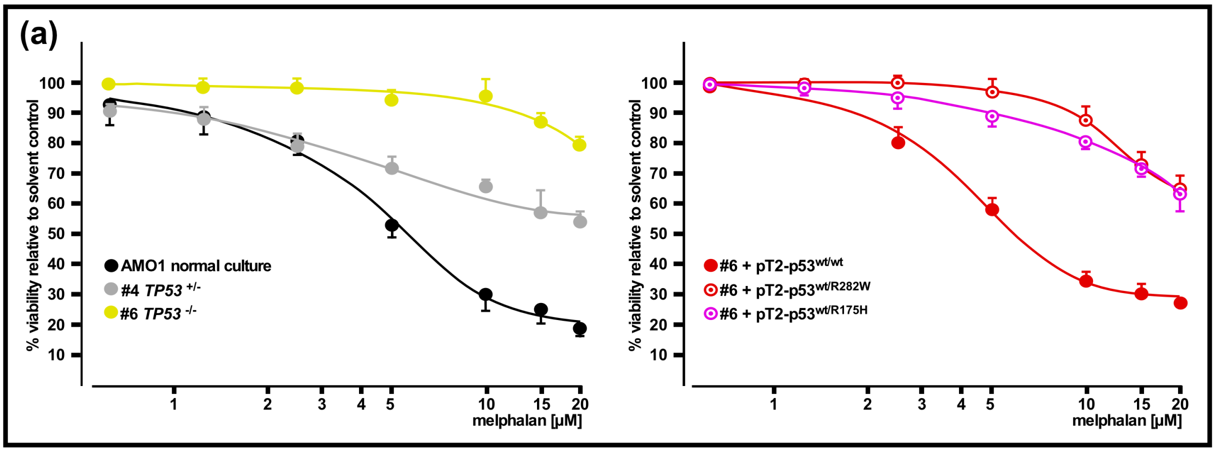

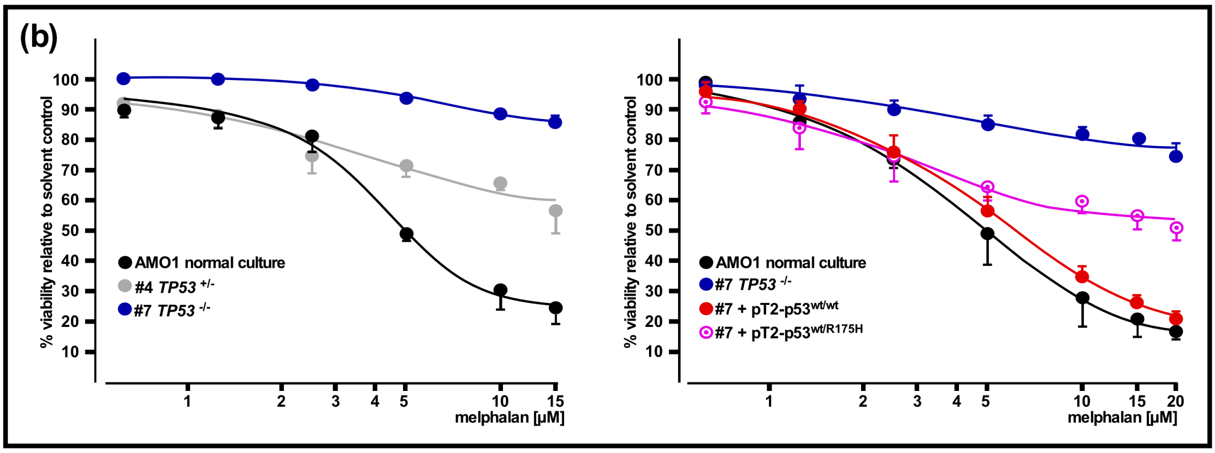

(c)

Annexin V survival assay (Figure 2c, left)

	Exp.1	Exp.2	Exp.3	Mean	SD	rel. to AMO-1 cell line
AMO-1 cell line	90,5	88,2	91	89,9	1,49	100
clone #7	91,3	88,4	91,3	90,33	1,67	100,5
clone #7 + $p53^{wt/wt}$	81,4	81,2	80,5	81,03	0,47	90,1
clone #7 + $p53^{wt/R282W}$	83,2	85,1	85,7	84,67	1,31	94,2
clone #7 + $p53^{R282W}$	93,3	91,8	94,1	93,07	1,17	103,4
clone #7 + $p53^{wt/R175H}$	89,1,	90,3	88,4	89,27	0,96	99,3
clone #7 + $p53^{R175H}$	89,4	89,8	88,9	89,37	0,45	99,4

alamarBlue viability assay (Figure 3b)

	Exp.1	Exp.2	Exp.3	Mean	SD
AMO-1 cell line	'100'	'100'	'100'	'100'	
clone #7	90,2	94,4	101,8	95,45	5,85
clone #7 + $p53^{wt/wt}$	94,5	88,8	95	92,75	3,44
clone #7 + $p53^{wt/R282W}$	106,6	92,7	104,8	101,35	7,57
clone #7 + $p53^{R282W}$	96,7	92,7	110,3	99,86	9,21

Supplementary Figure 3

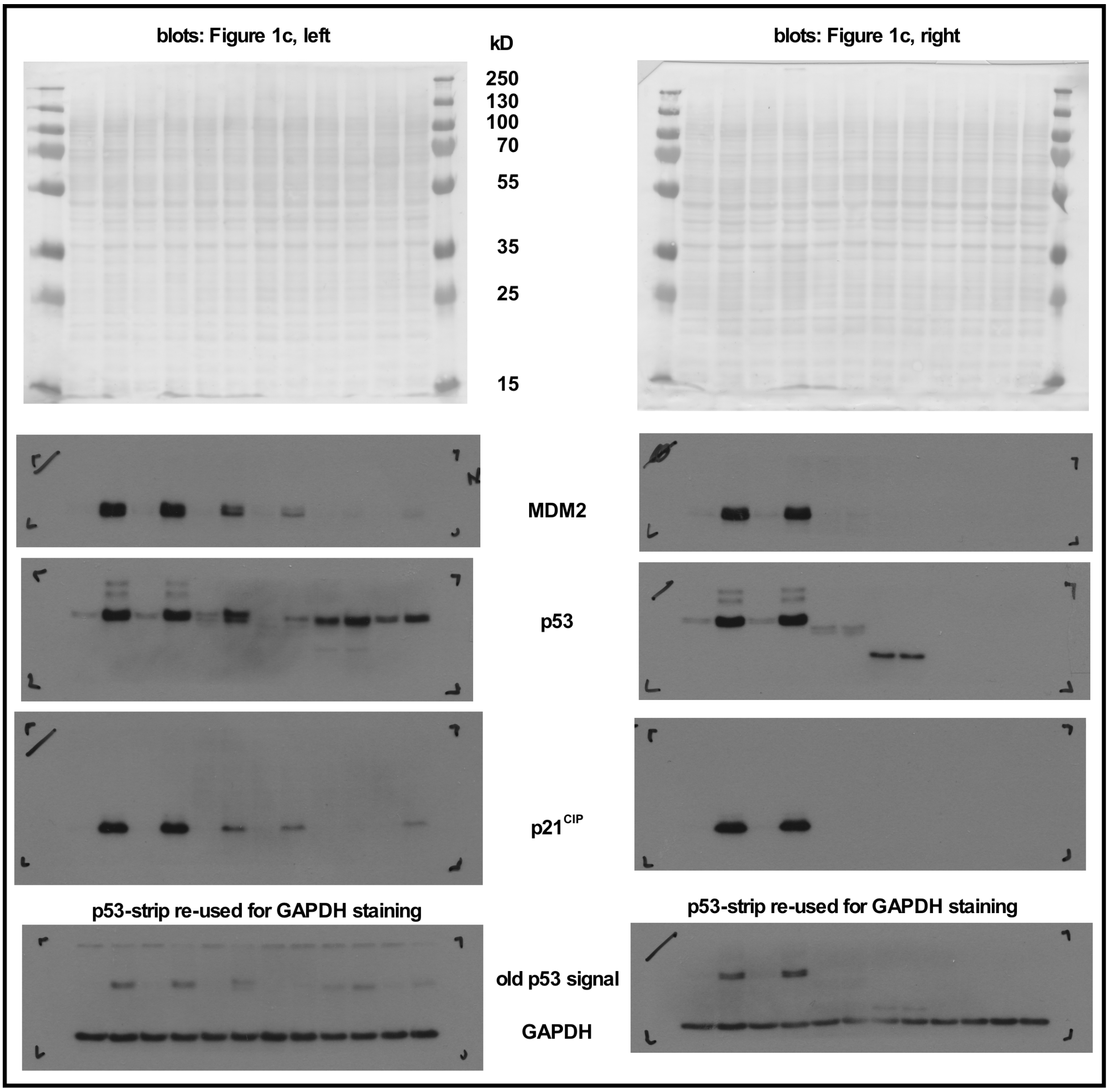

Supplementary Figure 4

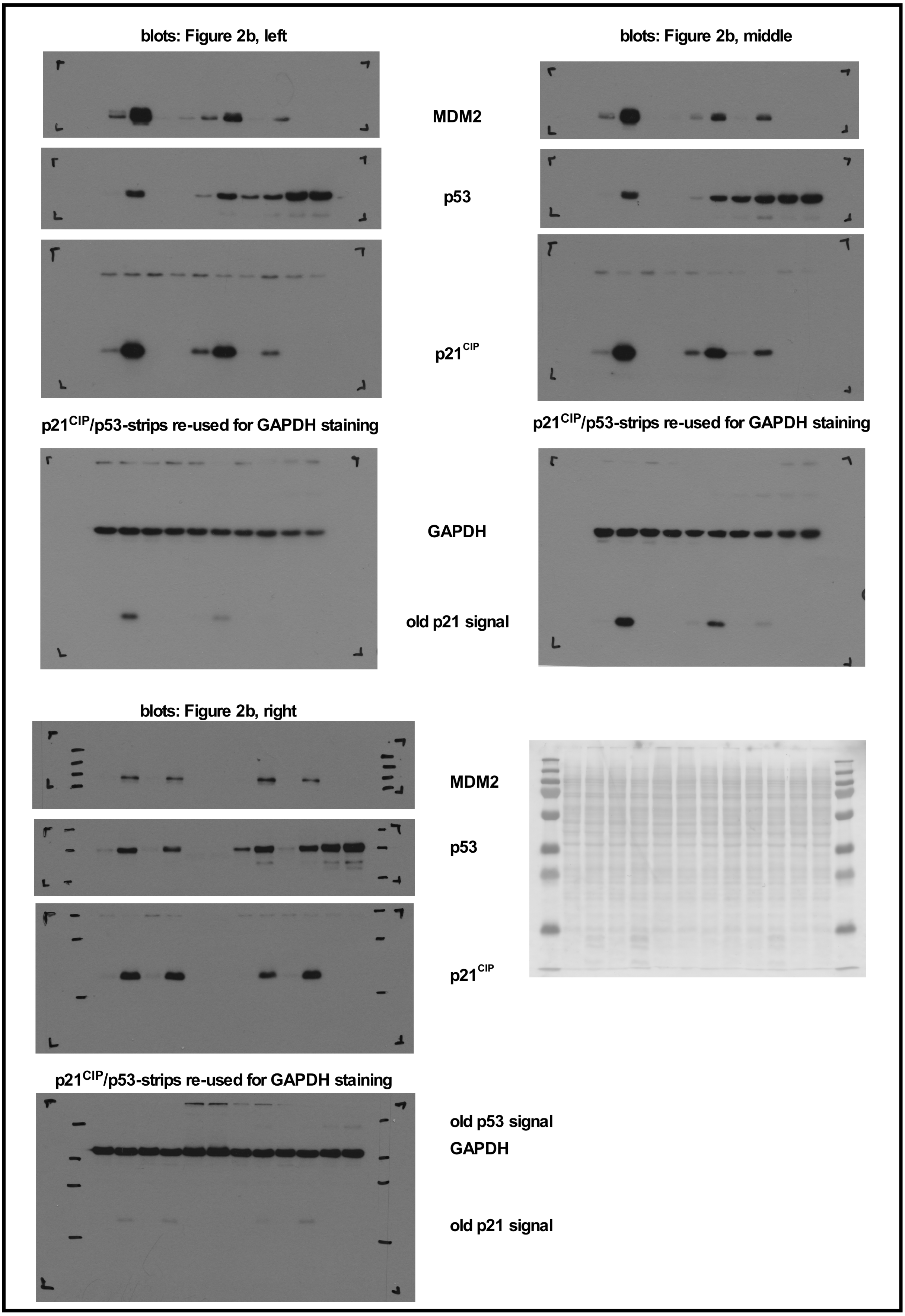

Supplementary Figure 5

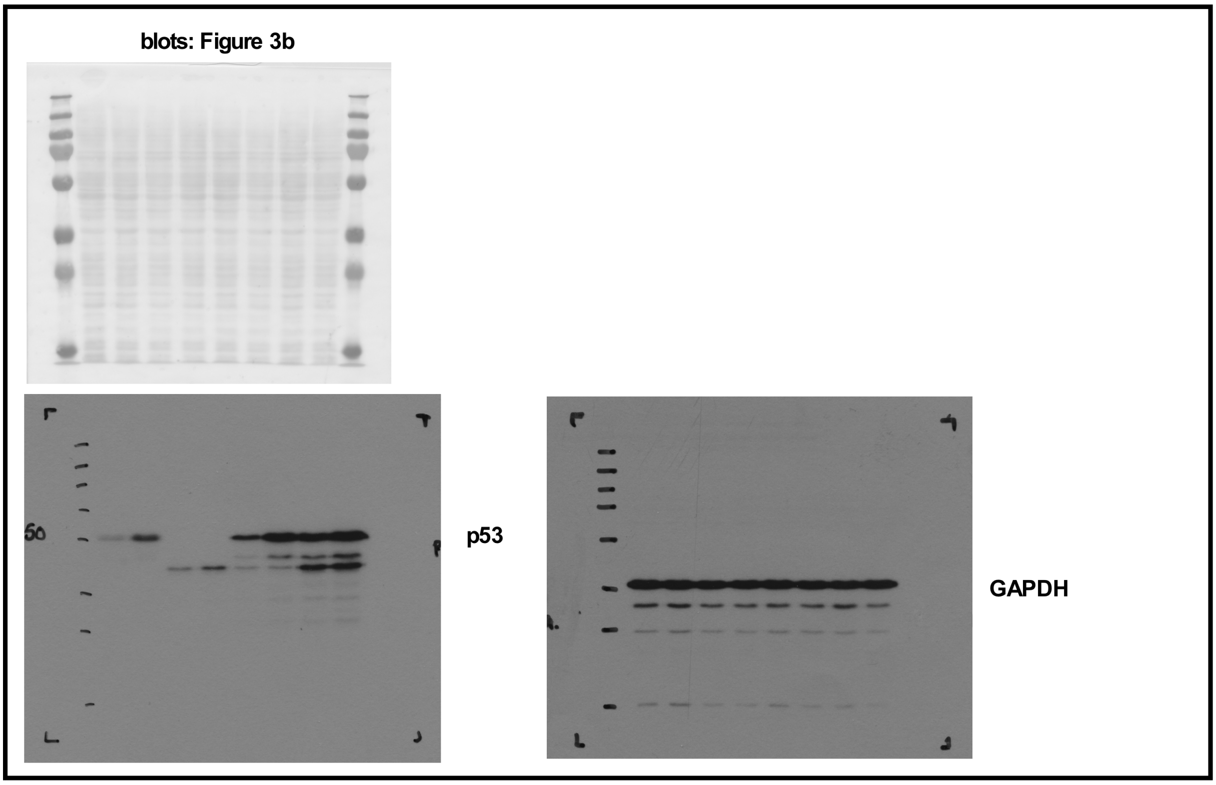

Supplementary Figure 6
